# Central Serous Chorioretinopathy Associated with Corticosteroid Use in a Patient with Leber Hereditary Optic Neuropathy: A Case Report

**DOI:** 10.3390/medicina61010007

**Published:** 2024-12-25

**Authors:** Lepsa Zoric, Aleksandra Petrovic, Vladimir Milutinovic

**Affiliations:** 1 School of Medicine, University of Pristina, 38220 Kosovska Mitrovica, Serbia; zoriclepsa@gmail.com; 2 Clinic for Eye Disease, University Clinical Center of Serbia, 11000 Belgrade, Serbia; dr.vladimir.milutinovic@gmail.com; 3 Health Center Dr Milutin Ivkovic, 11000 Belgrade, Serbia

**Keywords:** Leber hereditary optic neuropathy (LHON), central serous chorioretinopathy (CSCR), corticosteroid therapy

## Abstract

*Introduction*. Leber hereditary optic neuropathy (LHON) is a condition characterized by bilateral acute or subacute vision loss in seemingly healthy individuals. Depending on the disease stage and initial presentation, it is often diagnosed as optic neuritis. Elevated levels of endogenous and exogenous glucocorticoids have been associated with the onset of central serous chorioretinopathy (CSCR). In our patient, CSCR developed after only three days of pulse corticosteroid therapy, prescribed due to initial presentation as bilateral optic neuritis (papillitis). *Objective*. Through our case report, we aimed to highlight that CSCR can develop after the initiation of pulse corticosteroid therapy in a patient with LHON and to propose choroidal thickness as a potential contributing factor for this complication. *Case Presentation*. A 27-year-old male patient presented with painless subacute vision loss in both eyes. The decline in vision developed gradually over 20 days, prior to the patient’s referral to the UKCS Eye Disease Clinic for further examination and treatment, and was not accompanied by pain during eye movements. Initial investigations upon admission to the clinic established the diagnosis of optic neuritis. Consequently, pulse corticosteroid therapy was administered. Three days after the initiation of intravenous methylprednisolone, the patient developed bilateral central serous chorioretinopathy. After cessation of therapy, there was a rapid resolution of choroidopathy, but no improvement in visual acuity, prompting genetic testing. Subsequent laboratory results revealed a positive test for the LHON mutation m.3460 G>A (MT-ND1). *Conclusions*. LHON is often misdiagnosed as optic neuritis, as upon initial presentation the optic nerve disk often does not exhibit the apparent characteristics of LHON. Numerous studies have documented the development of central serous chorioretinopathy following corticosteroid treatment, though none have reported the onset of CSCR after only three days of pulse corticosteroid therapy. Increased choroidal thickness is a characteristic of the acute phase of LHON and may be associated with the development of CSCR in our patient.

## 1. Introduction

Leber’s hereditary optic neuropathy (LHON) is one of the most common hereditary optic neuropathies. It is a genetic disorder of mitochondrial DNA that clinically manifests as acute or subacute vision loss in seemingly healthy individuals. The disease typically results in complete blindness in both eyes, or less commonly in one eye [1].

LHON is caused by mutations in maternally inherited mitochondrial DNA.

LHON is currently associated with 33 point mutations in mitochondrial DNA [1]. The three most common mutations are m.11778 G>A, m.14484 T>G, and m.3460 G>A, which lead to damage in the mitochondrial respiratory chain [2,3]. Certain risk factors believed to additionally aggravate the mitochondrial energy production chain, and consequently accelerate the onset of LHON, include smoking and alcohol consumption. Thus, patients are advised to reduce alcohol intake and avoid smoking to minimize potential mitochondrial stress [1]. Additionally, nutritional deficiencies, such as vitamin B12 deficiency, may play a role in disease expression due to important metabolic cofactors [1]. Leber hereditary optic neuropathy primarily develops in men between the ages of 20 and 30, and less commonly in women. In atypical cases, it can occur between the ages of 10 and 60 [4].

The stages of progression of LHON are the asymptomatic, acute, dynamic, and chronic stages. A typical finding at the fundus during the acute phase of Leber hereditary optic neuropathy is peripapillary telangiectatic microangiopathy, tortuosity of blood vessels, and edema of the parapapillary nerve fiber layer [5,6]. Later, optic nerve atrophy occurs with focal degeneration of retinal ganglion cells and persistence of a centrocecal scotoma in the visual field, as well as an increase in choroidal thickness and accompanying thickening of the retinal nerve fiber layer (RNFL) [1]. Edema of the optic nerve head and peripapillary telangiectasia may or may not be observed in some patients initially [4].

In rare cases, although patients with the m.14484 T>G mutation may recover spontaneously, visual recovery is usually minimal [1,7].

LHON is often initially diagnosed as optic neuritis due to their similarity in clinical presentation [6].

Furthermore, central serous chorioretinopathy (CSCR) is a disease of the chorioretina characterized by serous detachment of the neurosensory retina, typically confined to the macular area. CSCR is considered a disease with multiple causes, all leading to similar abnormalities in choroidal blood vessels [8]. Additionally, changes in different retinal layers have been described, suggesting their varying involvement in this process [9].

Risk factors are numerous and include being of the male sex, choroidal thickness, hyperopia, corticosteroid use, type A personality [10], hypertension, gestational age at birth, Helicobacter pylori level, and rarely endocrine disorders (Cushing syndrome). Corticosteroids are known to potentially cause the onset of CSCR, as confirmed by numerous studies [8,11,12].

The methods used to assess our patient’s condition include neuro-ophthalmological examination, intraocular pressure measurement with an applanation tonometer, optical coherence tomography (OCT, Optovue Angiovue device), visual field testing (Humphrey field analyzer), and electrophysiology (Metrovision MonPack): Visual Evoked Potentials (VEP) and Pattern Electroretinography (PERG).

Through our case report, we aimed to highlight that CSCR can develop after the initiation of pulse corticosteroid therapy in a patient with LHON and to propose choroidal thickness as a potential contributing factor for this complication.

## 2. Case Report

A 27-year-old male patient was admitted to the Clinic for Eye Diseases at the University Clinical Center of Serbia (UKCS) in Belgrade due to vision loss in both eyes.

The patient reported a decline in vision in both eyes to his referring ophthalmologist 20 days prior to being referred to the UKCS for further examination and treatment. The decrease in visual acuity was painless, subacute, and unaccompanied by other neurological symptoms. Regarding other information in his personal and family history, the patient denied any relevant data at the time of admission. Also, the patient did not have any other medical conditions and was not on any medication.

Immediately upon admission, his best-corrected visual acuity was counting fingers at 5 m (0.08), and intraocular pressure was normal in both eyes. The intraocular pressure was measured using applanation tonometry and was 16 mmHg in the right eye and 15 mmHg in the left eye. Ocular motility was good. The anterior segment of both eyes was normal, although an afferent pupillary defect was noted, more pronounced in the left eye.

**Figure 1:**
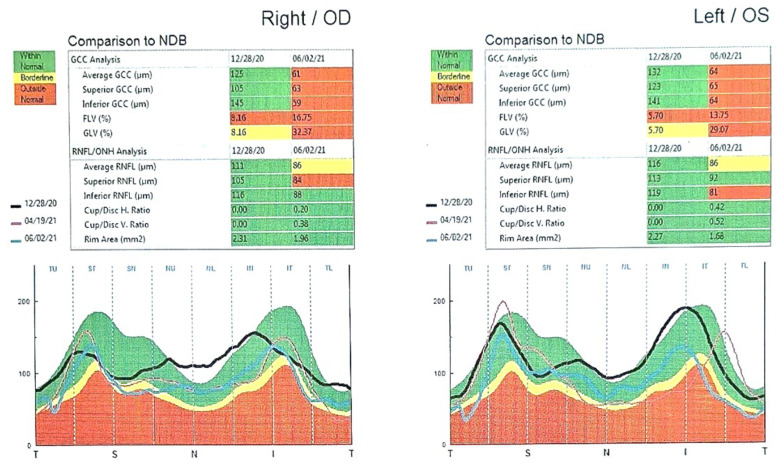
ONH/GCC OU—Focal and global loss of ganglion cells.

Fundoscopic examination revealed edema of the optic nerve head inferonasally in both eyes, normal blood vessel branching, and appearance. Thickening of nerve head fibers was confirmed by optical coherence tomography (OCT), where a slight focal and global loss of ganglion cells (FLV and GLV%) was also observed (Figure 1).

Computerized visual field testing showed a significant decrease in sensitivity, more pronounced in the upper regions of the visual field. Left eye: Visual Field Index (VFI) 68%, Mean Deviation (MD) −9.59 dB, Pattern Standard Deviation (PSD) 10.34 dB; right eye: VFI 73%, MD −10.98 dB, PSD 10.65 dB. The defect primarily affected the upper half of the visual field and a slightly central part of the inferior visual field.

Electrophysiological testing revealed reduced amplitudes and prolonged latencies of the P100 wave bilaterally, as well as a decrease in the PhNR wave in Photopic Negative Response Electroretinography (PhNRERG) and the n95 wave in and Pattern Electroretinography (PERG), with the n95/p50 ratio below 1.

Based on the clinical course and findings, and taking into account the vision loss in both eyes, the patient’s age, negative family history, and possible risk factors, the diagnosis of atypical optic neuropathy was made, likely inflammatory in nature. The patient was prescribed pulse corticosteroid therapy: intravenous methylprednisolone 1000 mg once daily for 5 days, along with gastroprotection with the use of proton pump inhibitors.

**Figure 2:**
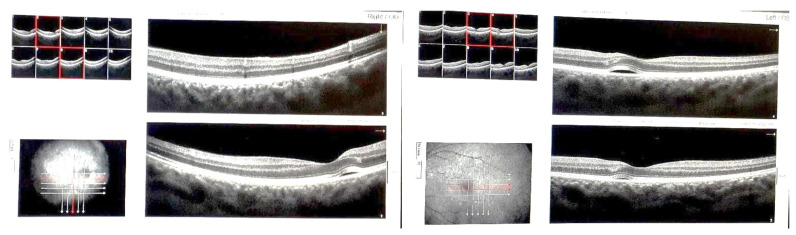
OCT of the macular region on the third day of corticosteroid therapy, showing CSCR—detachment of the neurosensory retina, with marginal thickening and hyperreflectivity of the outer nuclear layer and ellipsoid layer (**left**: OD, **right**: OS).

Three days after the initiation of intravenous methylprednisolone, the patient reported a further developing blurring of vision at a value of 3/60 in the right eye and 4/60 in the left eye (0.05 and 0.06). Examination revealed bilateral central serous chorioretinopathy (CSCR). OCT confirmed the development of CSCR in both eyes, with classic retinal elevation (Figure 2). Pattern Visual Evoked Potentials (PVEP) showed prolonged latencies bilaterally, while Flash Visual Evoked Potentials (FVEP) latencies and amplitudes were normal, with a lower amplitude in the right eye. PRERG showed reduced waves; PRERG showed reduced waves; ratio of n95:p50 was 1.38 on the right, while it was reduced to 0.67 on the left, with low percentages of accuracy, given the low visual acuity. A MERG61 bifixation was also performed, which showed a reduction in waves in all sectors, most pronounced in the center. The results of the new visual field testing were: right eye (OD) VFI 61%, MD −12.36 dB, PSD 12.19 dB; left eye (OS) VFI 74%, MD −10.52 dB, PSD 9.20 dB.

Corticosteroid therapy was gradually reduced over the next two days until it was eventually discontinued. Following the adjusted corticosteroid therapy, the treatment regimen was modified to include topical Trusopt (dorzolamide) two times a day in both eyes.

On optical coherence tomography (OCT), the beginning of reduction in Central Serous Chorioretinopathy (CSCR) was observed in the following days and was completely resolved by the time of the follow-up examination.

**Figure 3:**
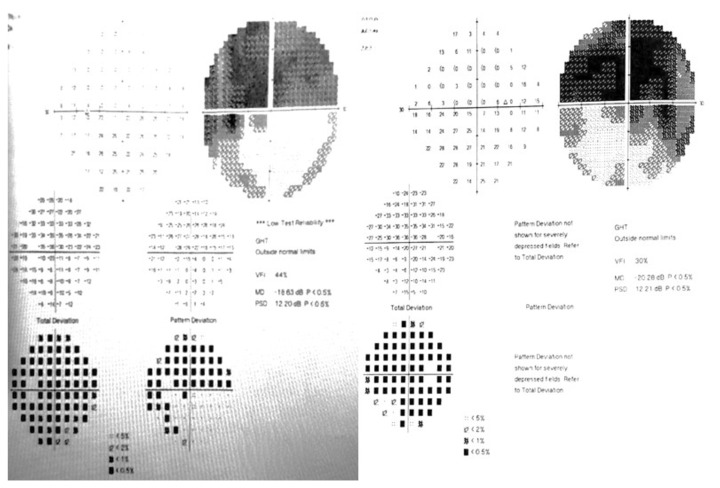
Computerized visual field three weeks after admission to the clinic (**left**: OS, **right**: OD).

Three weeks after admission to the clinic, visual field testing was repeated, revealing a decline in reference indicators: for the left eye (OS): Visual Field Index (VFI) 44%, Mean Deviation (MD) −18.63 dB, Pattern Standard Deviation (PSD) 12; and for the right eye (OD): VFI 30%, MD −20.28 dB, PSD 12.21 (Figure 3).

The patient was tested for possible causes of optic neuropathies: complete blood count and biochemistry, including lipid and anticoagulant status, antibodies to Herpes Simplex Virus (HSV), Varicella Zoster Virus (VZV), Cytomegalovirus (CMV), Epstein–Barr Virus (EBV), Hepatitis B Virus (HBV), Hepatitis C Virus (HCV), and Human Immunodeficiency Virus (HIV). Additional tests included the Quantiferon TB Gold test, antibodies to *B. burgdorferi*, *Treponema pallidum*, *Toxoplasma gondii*, the Venereal Disease Research Laboratory (VRDL), antibodies against myelin oligodendrocyte glycoprotein (Anti-MOG), antibodies against aquaporin-4 (Anti-AQP4) in serum, investigation of congenital and acquired thrombophilia, antinuclear antibodies (ANA), anti-neutrophil cytoplasmic antibodies (ANCA), rheumatoid factor (RF), and human leukocyte antigen (HLA) typing to exclude possible Vogt–Koyanagi–Harada syndrome [13] and angiotensin-converting enzyme (ACE).

Further investigations included MRI of the brain and cervicothoracic spinal cord, as well as internal medicine, cardiology, and neurologic examinations.

Fluorescein angiography was not performed due to the patient’s negative reaction (fear, collapse).

The results of the conducted tests did not indicate a possible etiology.

Due to the dynamic clinical presentation and negative test results, we conducted genetic testing for Leber hereditary optic neuropathy.

After several weeks, the results of genetic testing were obtained—mutation m.3460 G>A (MT-ND1). This confirmed the suspicion of LHON.

After three and a half months, the OCT findings showed atrophy of the ganglion cell complex, with RNFL atrophy more pronounced inferiorly and temporally in the left eye (Figure 1). The visual field was perimetrically non-functional, and visual acuity in the right eye was 0.10/60 (0.001), while in the left eye, it was L+P+.

The patient was informed about the available therapeutic options, including idebenone, and the process of categorization of blind and visually impaired individuals was initiated. The patient has voluntarily opted out of further observation.

## 3. Discussion

Among the risk factors for the development of Central Serous Chorioretinopathy (CSCR), the influence of corticosteroids is well known. In 1966, Jain and Singh described a case of CSCR that occurred during steroid therapy prescribed for the treatment of Reiter’s syndrome, which resolved after the therapy was discontinued [8]. Several subsequent reports of similar cases have shown a connection between the reduction or cessation of systemic corticosteroids and the resolution of CSCR [8]. Typically, CSCR occurs during prolonged corticosteroid use, most commonly with oral Prednisone for various conditions. There have been reported cases of CSCR following oral, nasal, dermatological, intravenous (IV), and intramuscular (IM) administration of corticosteroids [11,12]. However, it is very rarely observed during IV pulse corticosteroid therapy, as was the case with our patient.

The exact mechanism underlying this disorder has not yet been established. However, corticosteroids are believed to alter choroidal microcirculation by increasing platelet aggregation, leading to the subsequent formation of microthrombi and increasing blood viscosity [14].

Recent studies have also shown that corticosteroid use has long-term effects on the choriocapillaris in terms of increased permeability [15,16].

It is also known that CSCR is characterized or conditioned by increased thickness of the choroid at the posterior pole. Increased choroidal thickness is thought to be related to damage to choroidal vasculature and hyperpermeability of the choriocapillaris [11].

Vascular changes have also been noted in Leber’s Hereditary Optic Neuropathy (LHON), initially manifesting as angiopathy and the appearance of telangiectasia. In the chronic phase, the attenuation of choroidal blood vessels occurs. The pathophysiology of these changes is still under investigation. Currently, in both the asymptomatic and acute phases, a proliferation of mitochondria in endothelial and smooth muscle cells has been observed. This proliferation is responsible for the increased thickness of these blood vessels and for the narrowing of their lumen [6,17].

In addition, studies have also shown that the thickness of the macular and peripapillary choroid is increased in individuals with LHON during the acute phase of the disease, while thinning occurs in later stages [17].

Considering that increased choroidal thickness represents a common pathological finding in the acute phase of both LHON and CSCR, one might question whether these diseases share some common pathophysiological mechanisms. In this case, the question is raised whether it is justified to consider that CSCR, developed just three days after the initiation of pulse corticosteroid therapy, is precisely due to the presence of already compromised choroidal vasculature within the context of LHON.

## 4. Conclusions

A common characteristic of the acute phase of LHON and CSCR is increased choroidal thickness, although objective constraints (EDI OCT) placed a limitation in this case and did not allow for choroidal thickness measurement.

It can be suggested and inferred, as described in the literature, that the changes in choroidal morphology in the macular and peripapillary regions during the early stages of LHON may have contributed to the early onset of CSCR in our patient receiving corticosteroid therapy.

## Data Availability

All data can be obtained from the corresponding author upon request.
